# Body mass index and risk of dementia: Analysis of individual-level data from 1.3 million individuals

**DOI:** 10.1016/j.jalz.2017.09.016

**Published:** 2018-05

**Authors:** Mika Kivimäki, Ritva Luukkonen, G. David Batty, Jane E. Ferrie, Jaana Pentti, Solja T. Nyberg, Martin J. Shipley, Lars Alfredsson, Eleonor I. Fransson, Marcel Goldberg, Anders Knutsson, Markku Koskenvuo, Eeva Kuosma, Maria Nordin, Sakari B. Suominen, Töres Theorell, Eero Vuoksimaa, Peter Westerholm, Hugo Westerlund, Marie Zins, Miia Kivipelto, Jussi Vahtera, Jaakko Kaprio, Archana Singh-Manoux, Markus Jokela

**Affiliations:** a Department of Epidemiology and Public Health, University College London, London, UK; b Clinicum, Department of Public Health, Faculty of Medicine, University of Helsinki, Helsinki, Finland; c Finnish Institute of Occupational Health, Helsinki and Turku, Finland; d Centre for Cognitive Ageing and Cognitive Epidemiology, Alzheimer Scotland Dementia Research Centre, University of Edinburgh, Edinburgh, UK; e School of Social and Community Medicine, University of Bristol, Bristol, UK; f Institute of Environmental Medicine, Karolinska Institutet, Stockholm, Sweden; g Centre for Occupational and Environmental Medicine, Stockholm County Council, Stockholm, Sweden; h School of Health Sciences and Welfare, Jönköping University, Jönköping, Sweden; i Division of Epidemiology, Stress Research Institute, Stockholm University, Stockholm, Sweden; j Population-based Epidemiologic Cohort Unit, UMS 011, Inserm, Villejuif, France; k Department of Health Sciences, Mid Sweden University, Sundsvall, Sweden; l Department of Psychology, Umeå University, Umeå, Sweden; m Folkhälsan Research Center, Folkhälsan, Helsinki, Finland; n School of Health and Education, University of Skövde, Skövde, Sweden; o Turku University Hospital, Turku, Finland; p Institute for Molecular Medicine (FIMM), University of Helsinki, Helsinki, Finland; q Occupational and Environmental Medicine, Uppsala University, Uppsala, Sweden; r Department of Neurobiology, Karolinska Institute, Stockholm, Sweden; s National Institute for Health and Welfare, Helsinki, Finland; t University of Turku, Turku, Finland; u Institute of Behavioral Sciences, University of Helsinki, Helsinki, Finland

**Keywords:** Body mass index, Dementia, Cohort study, Bias, Obesity

## Abstract

**Introduction:**

Higher midlife body mass index (BMI) is suggested to increase the risk of dementia, but weight loss during the preclinical dementia phase may mask such effects.

**Methods:**

We examined this hypothesis in 1,349,857 dementia-free participants from 39 cohort studies. BMI was assessed at baseline. Dementia was ascertained at follow-up using linkage to electronic health records (N = 6894). We assumed BMI is little affected by preclinical dementia when assessed decades before dementia onset and much affected when assessed nearer diagnosis.

**Results:**

Hazard ratios per 5-kg/m^2^ increase in BMI for dementia were 0.71 (95% confidence interval = 0.66–0.77), 0.94 (0.89–0.99), and 1.16 (1.05–1.27) when BMI was assessed 10 years, 10-20 years, and >20 years before dementia diagnosis.

**Conclusions:**

The association between BMI and dementia is likely to be attributable to two different processes: a harmful effect of higher BMI, which is observable in long follow-up, and a reverse-causation effect that makes a higher BMI to appear protective when the follow-up is short.

## Highlights

• Data from 1.3 million adults from 39 prospective cohort studies were pooled for the analyses.
• Higher BMI was associated with increased risk of dementia when the follow-up was long.
• When follow-up was short, lower BMI was linked to increased dementia risk probably due to reverse causation.

## 1 Introduction

The costs of dementia are enormous and increasing globally [1]. Current clinical guidelines for dementia prevention view obesity as one of the modifiable risk factors [2], [3], but the evidence is based on a relatively limited number of observational studies and the findings are mixed [4], [5], [6], [7], [8], [9], [10], [11], [12]. The most recent meta-analysis, including 4 studies and 16,282 participants, suggested a 1.4-fold increased risk of dementia in the obese [9]. The largest study in the field, published after the inclusion date for the meta-analysis, found no increase in dementia incidence among the obese [13]. On the contrary, higher body mass index (BMI) was linked to lower dementia risk.

**Fig. 1:**
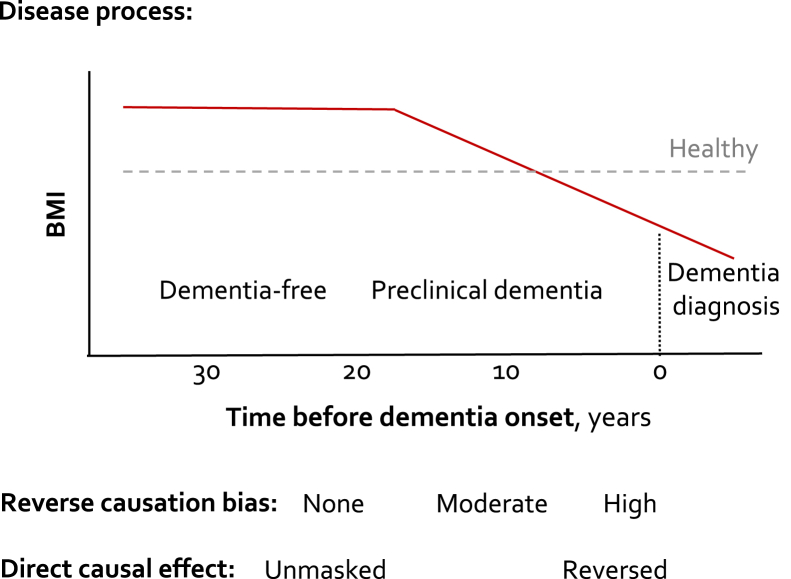
Conceptual model: Effect of reverse causation (preclinical disease reduces weight) on BMI at different etiological periods before dementia diagnosis. Abbreviation: BMI, body mass index.

The reasons for this discordance in findings are unclear. One possibility is that the observed association between BMI and dementia is attributable to two processes: one is a direct association between higher BMI and increased dementia risk, and the other is an association confounded by weight loss during the preclinical dementia phase, which leads a harmful exposure to appear protective via reverse causation (Fig. 1). This hypothesis is supported by the fact that clinical diagnosis of dementia is often preceded by a long (20–30 years) preclinical phase [14], [15], [16], [17] during which cardiometabolic changes, including weight loss, are common [5], [6], [18], [19]. Thus, lower BMI close to dementia onset might be a consequence of preclinical disease rather than a cause of dementia. The investigations [4], [5], [6], [7] supporting this two-process hypothesis are based on small numbers (N < 3000) and thus vulnerable to random errors. A further limitation is that these studies did not directly seek to determine the etiological phase at exposure measurement by stratifying the analyses by the length of follow-up between the assessment of BMI and dementia onset.

The purpose of the present analyses was to investigate the BMI-dementia association using raw unpublished data from over 1.3 million adults from Europe, the United States, and Asia. To separate direct and biased associations, we stratified the analysis by duration of follow-up. We assumed that BMI is little affected by preclinical dementia when the BMI assessment is long before dementia onset and is considerably affected when BMI is assessed nearer the diagnosis.

## 2 Methods

### 2.1 Study population

We searched the Individual-Participant Data Meta-analysis in Working Populations (IPD-Work) consortium [20], [21], the Inter-University Consortium for Political and Social Research (www.icpsr.umich.edu/icpsrweb/ICPSR), and the UK Data Service (http://ukdataservice.ac.uk) to identify eligible large-scale prospective cohort studies for which data on BMI and dementia were available. We included 39 prospective cohort studies from Europe, the United States, and Asia (Appendix 1), which comprised a total of 1,349,857 participants with no history of dementia; were population based with BMI assessed from all participants before the ascertainment of dementia; recorded hospital-treated dementia or dementia deaths; and had accrued a minimum of 3 years of follow-up.

### 2.2 Measurements

Height and weight at baseline were measured in 11 studies and self-reported in 28 (Appendix 1, avaliable in the online Supplementary Materials). BMI was calculated as weight in kilograms divided by height in meters squared. We assessed the following baseline covariates because they are known to be associated with BMI and dementia risk: education/socioeconomic position (harmonized into high, intermediate, and low), smoking (current smoker vs. other), and prevalent cardiometabolic disease (coronary heart disease, stroke, and diabetes; one or more vs. none) [2], [3].

We obtained information about dementia status at follow-up from national death and hospital admission registries, reimbursements for medical treatment of dementia, and surveys on physician-diagnosed diseases, although the exact definition varied between the studies (Appendix 1). We used all underlying diagnoses in electronic medical records. In line with the National Health Service's recommendations, dementias were denoted by International Classification of Diseases (10th revision) codes ICD-10 F00, F01, F03, G30, and G31 [22], [23].

### 2.3 Statistical analysis

We used a two-step individual-participant data meta-analysis including study-specific analyses in the first step and pooling the study-specific estimates in the second. In each study, we performed Cox regression to generate hazard ratios and accompanying 95% confidence intervals (CIs) for the association between BMI and dementia. In the National Health Interview Survey (NHIS), the National Health and Nutrition Survey (NHANES), and the Health and Retirement Study (HRS) [24], [25], [26], [27], appropriate sampling weights were used. As one study was based on twins [28], individual observations were not independent, and we therefore used Cox regression with cluster information to get robust standard error estimates applying Breslow's method for handling ties. In all studies, each participant was followed up from the date of BMI assessment to the first record of dementia, death, or the end of follow-up.

In the basic Cox model, BMI was included as a continuous variable to allow inclusion of studies with few dementia cases (minimum 10). As in previous large-scale pooled analyses [29], [30], [31], hazard ratios were expressed per 5-unit (kg/m^2^) increase in BMI, which represents the increase in relative risk associated with moving up one BMI category (e.g., from healthy weight to overweight or from overweight to obese). Age at BMI assessment, sex, and ethnicity were included as covariates. Additional adjustments were undertaken for education/socioeconomic status, smoking, and prevalent cardiometabolic disease.

To study reverse causation bias, we excluded the first years of follow-up [32], using a standard procedure in epidemiology, which involves increasing the length of time (follow-up) between measurement of the exposure and the outcome, thus reducing the likelihood that BMI levels are affected by preclinical dementia. Initially, we excluded the first 5 years of follow-up and then progressively increased the exclusion period in 5-year increments up to an exclusion period of 20 years. We expected these analyses to provide estimates increasingly less affected by reverse causation. In a second set of analyses, we examined the BMI-dementia association by follow-up time. To ensure sufficient numbers in these stratified analyses, we divided follow-up time into three 10-year categories: <10 years, 10–20 years, and >20 years after baseline. The first category includes BMI measurements during the preclinical phase of dementia, whereas in the last category, most BMI assessments are likely to be before this stage.

Studies with dementia death as outcome are vulnerable to ascertainment bias if the exposure (BMI) is associated with survival. For example, participants who died before the age of late-onset dementia (65 years) have less opportunity to receive a diagnosis of dementia than those who live longer. In contrast, participants dying around the median age of dementia recording (85 years in this study), have increased opportunities to be diagnosed with dementia. To study this bias, we examined whether BMI was associated with mortality before the age of 65 years and mortality after the age of 85 years [33]. In addition, we repeated the main analyses excluding dementia cases ascertained from death registries only.

We used SAS (version 9.4) or Stata (MP version 13.1) to analyze the study-specific data. In the second step of the analysis, we used meta-analysis to combine study-specific estimates. To provide conservative estimates and take into account that the associations are not necessarily similar across cohort studies, we present the summary hazard ratios from the random-effect analysis. We examined heterogeneity of the study-specific estimates with the *I*^*2*^ statistic (higher values denote greater heterogeneity). We used Stata (MP, version 13.1) to compute the meta-analysis.

## 3 Results

Descriptive characteristics for each cohort study are provided in Appendix 1. The weighted mean follow-up across studies was 16.1 years and ranged from 4.3 to 37.7 years. Over the 21,798,141 person-years at risk in 1,349,857 participants, 6894 incident dementia cases were recorded. Overall, we observed an inverse association between BMI and dementia. The age-, sex-, and ethnicity-adjusted hazard ratio per 5-unit increase in BMI was 0.87 (95% CI = 0.82–0.93). An *I*^*2*^ statistic of 65.9% indicated significant heterogeneity in the study-specific hazard ratios (*P* < .0001). To further examine this association, we undertook four sets of analyses.

### 3.1 Incremental exclusions to the follow-up

**Fig. 2:**
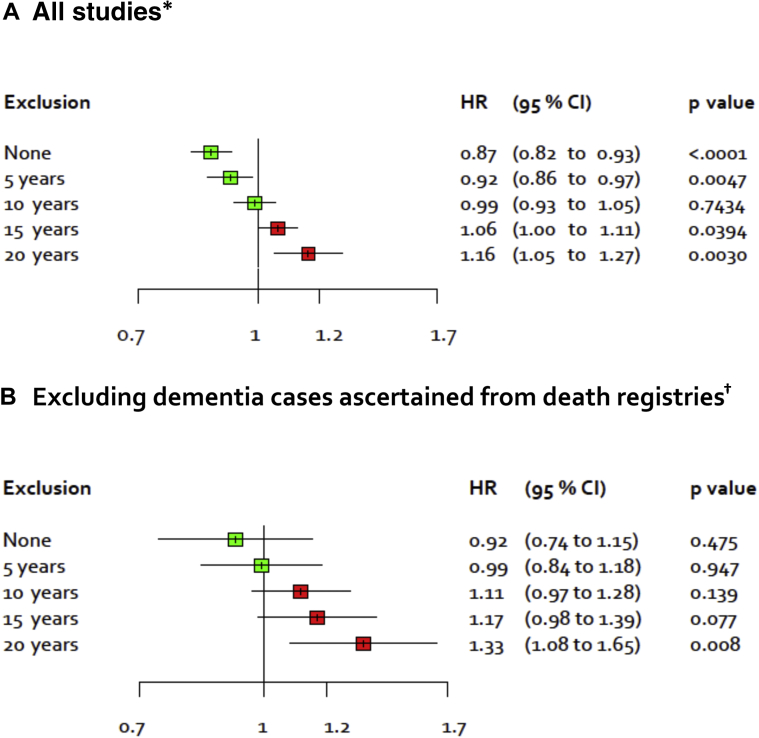
Age-, sex-, and ethnicity-adjusted HR for incident dementia per 5-kg/m^2^ increase in BMI after progressive exclusion of the follow-up period in all studies (A)^∗^ and in studies with dementia ascertainment using dementia morbidity data (B)^†^. ^∗^39 studies, total N = 1,349,857. ^†^5 studies, total N = 95,851. The figure shows that risk of bias due to preclinical dementia decreases with increasing exclusion of the follow-up period. Abbreviations: BMI, body mass index; CI, confidence interval; HR, hazard ratio.

We performed a detailed analysis of incremental exclusions to the follow-up period (Table 1). To assess BMI before the preclinical phase, we excluded up to 20 years of the follow-up (the numbers of participants and dementia cases in these analyses are shown in Appendix 1). With the progressive exclusions of follow-up, the magnitude of the BMI-dementia association changed in a stepwise manner such that the hazard ratio for higher BMI and reduced dementia risk was first attenuated to the null and then increased significantly above 1.0, suggesting higher BMI to be associated with greater risk of dementia (Fig. 2A, study-specific estimates in Appendix 2). The age-, sex-, and ethnicity-adjusted hazard ratios per 5-unit increase in BMI were 0.87, 0.92, 0.99, 1.06, and 1.16 after excluding the first 0, 5, 10, 15, and 20 years of follow-up. The last estimate, which provides evidence of an increased risk of dementia associated with higher BMI in models excluding the first 20 years of follow-up, was robust to further adjustments for potential confounders, including education/socioeconomic status, smoking, and cardiometabolic disease at baseline (Table 2). Furthermore, in these analyses, there was no evidence of heterogeneity in the study-specific effect estimates. Galbraith plots in Appendix 3 confirmed that no single study or studies drove these associations.

**Table 1:** Numbers and mean ages for the analytic samples

Exclusion of follow-up	Number of studies	N (total)	N (dementia cases)	Mean age at BMI assessment among dementia cases	Mean age at dementia diagnosis	Difference between mean ages at dementia and at BMI assessment
None	39	1,349,857	6894	69.1	84.3	15.2
First 5 years	34	1,212,806	6304	68.3	84.4	14.1
First 10 years	26	990,645	4924	66.3	84.8	18.5
First 15 years	15	694,223	3088	63.2	85.1	21.9
First 20 years	10	393,192	1499	58.4	84.8	26.4

Abbreviation: BMI, body mass index.

**Table 2:** Association between BMI and dementia risk after serial adjustments for potential confounders at baseline and exclusion of the first 20 years of follow-up∗

Adjustment in addition to age, sex, and ethnicity	Hazard ratio per 5 kg/m^2^ increase in BMI (95% confidence interval)	*P* value
None	1.17 (1.07–1.22)	.0003
Education/socioeconomic status	1.15 (1.06–1.24)	.0005
Smoking	1.17 (1.08–1.27)	<.0001
Cardiometabolic disease†	1.17 (1.07–1.27)	.0003
All the above	1.16 (1.07–1.25)	.0005

Abbreviation: BMI, body mass index.

∗ These analyses are based on eight studies with a follow-up longer than 20 years and data on covariates (total N = 147,581–147,893 depending on the model).

† Prevalent coronary heart disease, stroke, or diabetes at baseline.

**Fig. 3:**
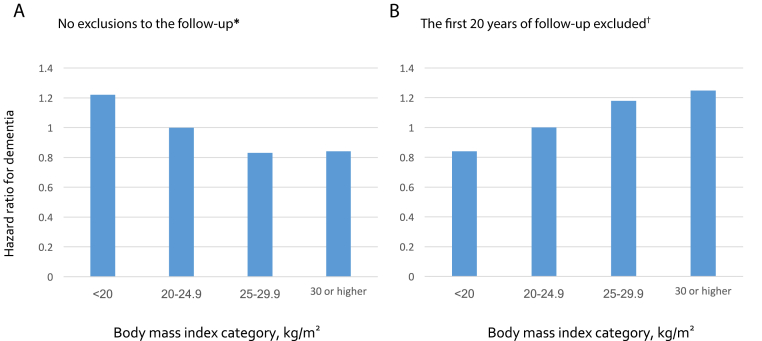
Shape of the association between BMI and dementia before (A) and after (B) exclusion of the first 20 years of follow-up. ^∗^39 studies, total N = 1,349,857. ^†^10 studies, total N = 391,596. The figure shows that risk of bias due to preclinical dementia is smaller after exclusion of follow-up.

We repeated the age-, sex-, and ethnicity-adjusted analyses using BMI categories (Fig. 3). Without exclusions of the follow-up period, the highest hazard ratio was observed for underweight (BMI < 20 kg/m^2^; 1.22, 95% CI = 1.06–1.41, *P* = .005), the lowest for obesity (BMI ≥ 30 kg/m^2^; 0.84, 95% CI = 0.75–0.94, *P* = .002), and overweight (BMI = 25–29.9 kg/m^2^; 0.83, 95% CI = 0.77–0.90, *P* < .0001). This ordering was reversed when we sought to reduce reverse causation bias by excluding 20 years of the follow-up.

Assuming that less biased effect estimates are observed after the exclusion of cases with BMI assessment close to dementia diagnosis (i.e., during the preclinical phase), the opposite associations before and after exclusions support the hypothesis that the BMI-dementia association is attributable to two processes, a direct effect and reverse causation.

### 3.2 Analysis stratified by follow-up time

**Fig. 4:**
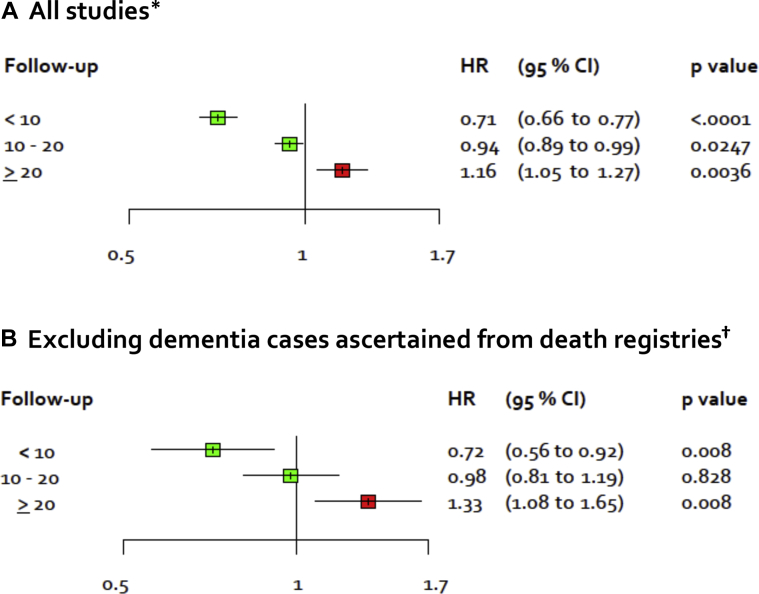
Age-, sex-, and ethnicity-adjusted hazard ratio for dementia per 5-kg/m^2^ increase in BMI in analysis stratified by follow-up period in all studies (A) and in studies with dementia ascertainment using dementia morbidity data (B). ^∗^39 studies, total N = 1,349,857. ^†^5 studies, total N = 95,851. The figure shows that risk of bias due to preclinical dementia is smaller at later follow-up periods. Abbreviations: BMI, body mass index; CI, confidence interval; HR, hazard ratio.

In the second set of analyses, we examined stratification by follow-up. Fig. 4A shows a forest plot for the association of BMI with dementia risk during the first 10 years, 10–20 years, and more than 20 years after baseline (study-specific results in Appendix 4). The corresponding age-, sex-, and ethnicity-adjusted summary hazard ratios were 0.71 (95% CI = 0.66–0.77, *P* < .0001), 0.94 (95% CI = 0.89–0.99, *P* = .02), and 1.16 (95% CI = 1.05–1.27, *P* = .004), respectively. The latter hazard ratio was 1.14 (95% CI = 1.04–1.25, *P* = .02) in studies with measured weight and height and 1.09 (95% CI = 0.99–1.19, *P* = .07) in studies with self-reported weight and height, suggesting that the result does not significantly vary by the method of BMI assessment. The increasing strength of the association between higher BMI and dementia as the length of follow-up increases and the diagnosis of dementia is increasingly distant from the assessment of BMI is consistent with the hypothesis that the harmful effect of a higher BMI becomes evident after removal of reverse causation bias.

### 3.3 Sensitivity analyses

We performed a series of sensitivity analyses with mortality as the outcome to examine potential ascertainment bias (Appendix 5). In most data sets included in this study, dementia cases included those ascertained using death certificates; thus, dementia could be ascertained only in participants who died. Higher baseline BMI was associated with higher overall mortality before the age of 65 years (when dementia is still rare) and lower mortality after 85 years (the median age of dementia diagnosis in this study). These findings suggest that survival bias, if anything, underestimates the status of high BMI as a risk factor.

To further examine the robustness of our findings, we repeated the main analyses after excluding 34 studies where dementia was ascertained only using death certificates. In total, 437 dementia cases in five studies (total N = 95,851) were ascertained using data other than death certificates. The pattern of results in the main analyses was replicated. In fact, the hazard ratios per 5-unit increase in BMI were slightly higher: 0.92, 0.99, 1.11, 1.17 and 1.33 after excluding the first 0, 5, 10, 15 and 20 years of follow-up (Fig. 2B; study-specific findings in Appendix 6). Higher effect estimates in Fig. 2B, corresponding to approximately one 5-year exclusion, are expected given that dementia is recorded first in morbidity data and only later in mortality data (Fig. 2A). In analysis stratified by duration of follow-up, the hazard ratio per 5-unit increase in BMI was 0.72 during the first 10 years of follow-up, 0.98 between 10 and 20 years, and 1.33 more than 20 years after baseline, again replicating findings in the main analysis (Fig. 4B, Appendix 6).

## 4 Discussion

In this collaborative study of over 1.3 million adults from Europe, the United States, and Asia, higher BMI was associated with increased dementia risk when weight was measured >20 years before dementia diagnosis, but this association was reversed when BMI was assessed <10 years before dementia diagnosis.

The findings of this study are consistent with the hypothesis that the BMI-dementia association is attributable to two processes: a direct (causal) effect and reverse causation as a result of weight loss during the preclinical dementia phase. Analyses stratified by duration of follow-up offered an approach to study this because BMI assessment long before dementia onset is more likely to reflect a causal process, whereas BMI assessment near dementia onset is likely to be biased by preclinical dementia. Consistent with this hypothesis, higher BMI increased dementia risk when weight was measured >20 years before dementia diagnosis (typically in midlife) but was associated with reduced risk when BMI was assessed <10 years before dementia diagnosis (i.e., typically in old age). This pattern of results was replicated in analyses serially excluding the first years of follow-up.

Our findings are in agreement with the most recent systematic review and meta-analysis, published in 2016, which identified four cohort studies with BMI assessed in midlife and incident dementia ascertained at older ages by clinical examinations at follow-up [9]. Although this is not a universal observation [34], [35], the summary relative risk of dementia for obesity compared with normal weight in midlife was significantly elevated. In the Cardiovascular Health Study of 2800 US adults, an increased risk of dementia was found among those obese in midlife but was reversed for late-life BMI [5], a finding replicated in the CAIDE study of 1300 Finnish adults [7]. In the Honolulu-Asian Aging Study of 2000 men, midlife BMI was nonsignificantly higher in individuals who subsequently developed dementia, but at older ages, they lost weight and had a lower BMI than those who remained free of dementia [4]. In the Gothenburg study of 1500 Swedish women, those who developed dementia had less increase in BMI from age 38 to 70 years than those who remained dementia free [6], [36]. Our findings, based on data from 1.3 million individuals from different regions of the world, add to this evidence by demonstrating the reversion of the BMI-dementia association as a function of decreasing distance between BMI assessment and dementia onset (and increasing impact of preclinical dementia).

Our use of electronic health records to ascertain dementia, like all methods, has some advantages and disadvantages. An advantage of this method is that there is virtually no loss to follow-up and thus selection bias arising from differential sample retention is avoided. For comparison, in studies that use repeated cognitive testing as part of dementia ascertainment, dropout may be as high as 40% at examination, leading to a significant cumulated sample attrition and therefore potential bias [9]. A disadvantage of electronic health records, however, is that they are unlikely to capture all dementia cases [37], [38]. In particular, studies where dementia outcomes are based on mortality data are not complete as they are based only on deceased patients and, even for them, dementia recording is delayed by the 3- to 9-year median lag between the clinical onset of dementia and death [39], [40]. Other concerns include the possibility that dementia as a cause of death is listed only among those who have experienced weight loss (“wasting”) before death as this would contribute to overestimation of a reverse causation effect.

As the present meta-analysis is based on a series of studies in which investigators ascertained dementia in different ways, we had the possibility to undertake a validation exercise. Thus, we repeated the main analyses excluding dementia status drawn from death certificates. The same pattern of results was evident as in the main analyses: higher BMI was associated with greater risk of dementia when BMI was measured many years before dementia onset, whereas an inverse relationship was apparent when BMI was measured closer to dementia ascertainment. In analyses exploring survival bias, we found that higher baseline BMI was associated with an increased risk of all-cause mortality before the age of 65 years but lower mortality risk after the age of 85 years (the median age of dementia diagnosis). These findings suggest that, compared with their normal weight counterparts, obese individuals were less likely to live long enough to develop dementia and more likely to die from conditions that are known to be related to increased dementia risk, such as diabetes and cardiovascular diseases [30], [31], [33], [41]. Given these findings, differences in survival may have contributed, if anything, to an underestimation of the strength of the association between BMI and dementia.

Stratification and progressive exclusion to the follow-up period to examine direct effect and reverse causation are, in effect, subgroup analyses, raising the question as to whether our findings could be an artifact of random variability as a result of reduced sample size. However, the consistent stepwise change in the BMI-dementia association at each additional exclusion of 5 years of the follow-up period in the main and supplementary analyses suggests that random variability is an unlikely explanation of our findings.

Taken together, these findings provide new evidence for the hypothesis that the association between BMI and dementia is attributable to two distinct processes; one of which is a harmful effect of higher BMI and the other reverse causation bias contributing to an inverse association between BMI and dementia. By dissecting these processes in stratified analyses, our study provides a plausible explanation for the inconsistencies in some of the prior studies on BMI and dementia. Further research is needed to examine underlying mechanisms for weight loss during the preclinical stage, including cognitive impairment leading to impaired self-care, reduced appetite due to decreased olfactory perception or changes in the regulation of satiety, and disturbed energy homeostasis. Future studies should also examine whether the role of BMI in dementia etiology varies between dementia subtypes, such as Alzheimer's disease, vascular dementia, frontotemporal dementia, and Lewy body dementia.

**Research in Context**

1. Systematic review: High body mass index (BMI) is suggested to increase the risk of dementia, but evidence is inconsistent. One hypothesis to explain this inconsistency is that weight loss during the preclinical phase of dementia biases the association, such that studies with a short follow-up, which assess BMI in late life, show that BMI has a protective association with dementia. However, few studies have examined the importance of length of follow-up and whether high BMI is more strongly related to dementia when assessed before preclinical dementia stage, that is, decades before dementia onset, than when assessed near dementia onset.
2. Interpretation: We explored the BMI-dementia association using raw, unpublished data from 1.3 million adults from Europe, the United States, and Asia. Our findings from analyses stratified by duration of follow-up lend support for a direct association between BMI and dementia and also provide some support for reverse causation. Higher BMI was associated with increased dementia risk when BMI was assessed more than 20 years before dementia diagnosis, but lower BMI predicted dementia when BMI was assessed less than 10 years before diagnosis.
3. Future directions: To avoid bias, the etiologic phase should be taken into account in studies examining dementia risk factors.

## Supplementary data

Appendices 1-6
