# Supplementary material for: Body mass index and risk of dementia: Analysis of individual-level data from 1.3 million individuals
Source: Alzheimers Dement. 2018 May;14(5):601–9. doi: 10.1016/j.jalz.2017.09.016 (PMC5948099; doi:10.1016/j.jalz.2017.09.016)
Supplement: Appendices 1-6 [file mmc1.pdf]

Appendices

	Page
**Appendix 1**<br>Description of the included cohort studies	2
**Appendix 2**<br>Forest plots for meta-analysis of dementia risk per 5-unit increment in BMI after incremental exclusions to the follow-up	8
**Appendix 3**<br>Galbraith plots for the association between BMI and dementia after gradual exclusions to follow-up	12
**Appendix 4**<br>Stratified meta-analysis: Forest plots for dementia risk per 5-unit increment in BMI by the duration of follow-up	14
**Appendix 5**<br>Forest plots for meta-analyses of mortality risk before age 65 and after age 65 per 5-unit increment in BMI	16
**Appendix 6**<br>Study-specific estimates for the association between BMI and dementia after excluding dementia cases ascertained from death registries.	18

Appendix 1

Description of the included cohort studies

Alameda County Study (Alameda), USA
The Alameda County Study is a prospective cohort study of a stratified random sample of Alameda County (California, USA) households with non-institutionalized residents, aged 21 and older, or aged 16-21 if married. The purpose of this survey was to explore the influence of health practices and social relationships on the physical and mental health of a typical sample of the population. The study started in 1965 with a sample of 6,928 respondents (including approximately 500 women aged 65 years and older). The 1974 questionnaire, which was used as the baseline year for the current study 6861participants had data on BMI and a dementia follow-up, and were thus eligible to this study.
Dementia was defined using death records *ICD 9 209-294, 331.0, 331.1, 331.2, 331.8, 331.9*
Reference: Berkman L, Breslow L. Health and ways of living: the Alameda County Study. New York, 1983.

Finnish Public Sector study (FPS), Finland
The Finnish Public Sector study is a prospective cohort study comprising the entire public sector personnel of 10 towns (municipalities) and 21 hospitals in the same geographical areas. Participants, who were recruited from employers' records in 2000-2002, were individuals who had been employed in the study organisations for at least six months prior to data collection. 48 592 individuals (9 337 men and 39 255 women aged 17 to 65) responded to the questionnaire. Of these, 46,880 had data on body mass index based on self-reported weight and health and were eligible for our meta-analyses. Ethical approval was obtained from the ethics committee of the Finnish Institute of Occupational Health. Participants were linked to drug reimbursement, hospitalisation and death registers. Dementia was defined using ICD-10, codes F00, F01, F02, F03, G30 and G31 (31.0, 31.1, 31.8)
Reference: Kivimäki M, Lawlor DA, Davey Smith G, et al. Socioeconomic position, co-occurrence of behavior-related risk factors, and coronary heart disease: the Finnish Public Sector study. *Am J Public Health* 2007; **97**: 874-9.

Finnish Twin Cohort (FTC) study, Finland
The older Finnish Twin Cohort study is a longitudinal study of all Finnish same-sex twins born before 1958. At the baseline in 1975, a postal questionnaire survey consisting of questions on health and health-related behaviors was conducted. The mean age of the participants was 36.3 (range 18.3 to 95.9) years at the start of follow-up May 1, 1976. Data for self-reported height and weight were available for 12,630 men and 13,184 women resident in Finland. Responses and returning completed questionnaire were considered as consent in postal questionnaire data collection in 1975; the study was approved by the National Board of Health. The use of the cohort for register follow-up studies has been approved by the Ethical committee of the Department of Public Health, University of Helsinki. Data and permission for use of cause of death data were obtained from Statistics Finland.

Dementia death was defined according to appropriate versions of the International Classification of Diseases (ICD). In ICD8 (deaths between 1970 and 1986) the following diagnoses were counted as a dementia death: Dementia senilis et praesenilis (ICD 290 category), Senilis (ICD 290,00), Morbus Alzheimer (ICD 290,01), Morbus Pick (ICD 290,11), Praesenilis alia sive NUD (ICD 290,19), Senilitas, psychosi non indicata (ICD 794), Marasmus senilis (ICD 794,00), and Senilitas alia sive NUD (ICD 794,09). In ICD9 (years 1987 to 1995) the following diagnoses were categorized as a dementia death: Dementia senilis et praesenilis (ICD 290 category, ICD 2900A), Morbus Alzheimer (ICD 3310A, Morbus Pick (ICD 3311A), Dementia e multis infarctibus (ICD 4378A), and Senilitas, psychosi non indicata (ICD 797). In the most recent period (years 1996 to 2011, ICD-10) the following diagnoses were considered to be a dementia death: Dementia vascularis (F01), Dementia non spesificata (F03), Morbus Alzheimer (G30), and Senilitas (R54). Both underlying and contributing codes were used.
References: Kaprio J, Koskenvuo M. Genetic and environmental factors in complex diseases: the older Finnish Twin Cohort. *Twin Res* 2002; **5**: 358-65; Iso-Markku P, Waller K, Kujala UM, Kaprio J. Physical activity and dementia: long-term follow-up study of adult twins. Ann Med. 2015; **47** :81-7.

Gazel, France
Gazel is a prospective cohort study of 20 625 employees (15 011 men and 5 614 women) of France's national gas and electricity company, Electricité de France-Gaz de France (EDF-GDF). Since the study baseline in 1989, when the participants were aged 35–50 years, they have been posted an annual follow-up questionnaire to collect data on health, lifestyle, individual, familial, social, and occupational factors. BMI was measured from self-reported height and weight in 1997 and 11 342 had data and were eligible for our meta-analysis. The GAZEL study received approval from the national commission overseeing ethical data collection in France (Commission Nationale Informatique et Liberté). Data from Gazel, WOLF-S, WOLF-N, and HeSSup were pooled to achieve sufficient case numbers (labelled IPD-Work other).
Dementia was defined using data from annual follow-up surveys requesting reported doctor-diagnosed demensis Alzheimer.
Reference: Goldberg M, Leclerc A, Bonenfant S, Chastang JF, Schmaus A, Kaniewski N, et al. Cohort profile: the GAZEL Cohort Study. *Int J Epidemiol* 2007; **36** :32-9.

Health and Lifestyle Survey (HALS)
UK HALS is a nationwide sample survey of community dwelling adults in England, Scotland, and Wales. In 1984/1985, a total of 12,254 addresses were randomly chosen from Electoral Registers and one adult aged 18 years or over was selected from each household. A total of 9003 adults participated in the baseline examination. Ethical approval for the main HALS surveys was received from the BMA Ethical Committee before the launch of survey. Participants were linked to mortality registers and deaths from dementia were defined using ICD 9 209-294, 331.0, 331.1, 331.2, 331.8, and 331.9.
Reference: Cox BD, Blaxter M, Buckle ALJ, et al. The Health and Lifestyle Survey: A Preliminary Report. London: Health Promotion Trust; 1987.

Health and Social Support (HeSSup), Finland

The Health and Social Support (HeSSup) study is a prospective cohort study of a stratified random sample of the Finnish population in the following four age groups: 20–24, 30–34, 40–44, and 50–54. The participants were identified from the Finnish population register and posted an invitation to participate, along with a baseline questionnaire, in 1998. A total of 16 666 had data on BMI from self-reported height and weight and were thus eligible for our meta-analyses. The Turku University Central Hospital Ethics Committee approved the study. Data from Gazel, WOLF-S, WOLF-N, and HeSSup were pooled to achieve sufficient case numbers (labelled IPD-Work other).

Participants were linked to drug reimbursement, hospitalisation and death registers. Dementia was defined using ICD-10, codes F00, F01, F02, F03, G30 and G31 (31.0, 31.1, 31.8)

Reference: Korkeila K, Suominen S, Ahvenainen J, Ojanlatva A, Rautava P, Helenius H, et al. Non-response and related factors in a nation-wide health survey. *Eur J Epidemiol* 2001; **17**: 991-9.

Health and Retirement Study (HRS), USA

The Health and Retirement Study (HRS) is a leading source for information on the health and economic well-being of adults age 50 and older in the United States. The HRS is a longitudinal project sponsored by the National Institute on Aging and the Social Security Administration. The first cohort was interviewed in 1992. Data on height, weight plus dementia follow-up were available for 9,899 men and women.

Participants were linked to mortality registers. Deaths from dementia were defined using ICD 10 G30 (Alzheimer's disease).

References: Growing Older in America: The Health and Retirement Study. Available at http://hrsonline.isr.umich.edu/index.php?p=dbook. June 25, 2016.

Juster FT, Suzman R. An overview of the Health Retirement Study. J Human Resources 1995;30(suppl): S7–S56.

Webpage: http://hrsonline.isr.umich.edu

National Health and Nutrition Survey (NHANES) 1971, 1976, 1988, 1999, 2001, 2003, 2005 and 2007, USA

The National Health and Nutrition Examination Survey (NHANES) is a programme of studies designed to assess the health and nutritional status of adults in the United States. The original NHANES (original) sample included 20,729 persons 25 to 74 years of age. NHANES 1971 (original, n = 14 396), 1976 (n=9,246), 1988 (n=18,072), 1999 (n=4,900), 2001 (n=5,223), 2003 (n =5,189), 2005 (n=5,234) and 2007 (n=5,884) are independent prospective cohort studies with height and weight measurement at baseline and a follow-up for cause-specific deaths.

Available ICD codes for dementia as a cause of death varied between study baseline: For NHANES 1971, 1976 and 1988, dementia deaths were defined using ICD 9 codes: *209-294, 331.0, 331.1, 331.2, 331.8, 331.9.* For NHANES 1999 to 2007, dementia deaths were defined using ICD 10 G30 (Alzheimer's disease).

References: Madans JH, Cox CS, Kleinman JC, et al. 10 years after NHANES I: mortality experience at initial follow up, 1982-84. *Public Health Rep* 1986; **101**: 474-81.

Flegal KM, Carroll MD, Kit BK, Ogden CL. Prevalence of obesity and trends in the distribution of body mass index among US adults, 1999-2010. *JAMA* 2012; **307**: 491-7.

Webpage: http://www.cdc.gov/nchs/nhanes/index.htm

National Health Interview Survey (NHIS) 1986-2009, USA

The National Health Interview Survey (NHIS) is a programme of studies monitoring the health of the U. S. residents since 1957. NHIS data on a broad range of health topics are collected through personal household interviews. The U.S. Census Bureau is the data collection agent for NHIS. For self-reported weight and height at baseline with a sufficient mortality follow-up for dementia deaths, data are publicly available from surveys in 1986 (n=43,028), 1987 (n=85,100), 1988 (n=85,140), 1989 (n=81,345), 1990 (n=83,459), 1991 (n=83,102), 1992 (n=88,060), 1992 (n=75,781), 1993 (n=75,781), 1994 (n=79,291), 1995 (n=68,721), 1996 (n=42,239), 1997 (n=33,523), 1998 (n=29,768), 1999 (n=28,178), 2000 (n=29,481), 2001 (n=30,218), 2002 (n=27,736), 2003 (n= 27,153), 2004 (n=28,187), 2005 (n=28,053), 2006 (n=22,312), 2007 (n=21,552), 2008 (n=20,502), and 2009 (n=26,470). Dementia was defined using death records, ICD-9 codes 209-294, 331.0, 331.1, 331.2, 331.8, and 331.9 or ICD-10 code G30 (Alzheimer's disease).

Reference: Dawson DA. Ethnic-differences in female overweight - data from the 1985 National-Health Interview Survey. *Am J Public Health* 1988; **78**: 1326-29.

Webpage: http://www.cdc.gov/nchs/nhis/

WOLF (Work, Lipids, and Fibrinogen) Stockholm and WOLF Norrland studies, Sweden

The WOLF (Work, Lipids, and Fibrinogen) Stockholm study is a prospective cohort study of 5673 people aged 19-70 and with data on BMI from measured height and weight working in companies in Stockholm county. WOLF Norrland is a prospective cohort of 4708 participants with data on BMI aged 19-65 working in companies in Jämtland and Västernorrland counties. At study baseline the participants underwent a clinical examination and completed a set of health questionnaires. For WOLF Stockholm, the baseline assessment was undertaken at 20 occupational health units between November 1992 and June 1995 and for WOLF Norrland at 13 occupational health service units in 1996-98. The Regional Research Ethics Board in Stockholm, and the ethics committee at Karolinska Institutet, Stockholm, Sweden approved the study. Data from Gazel, WOLF-S, WOLF-N, and HeSSup were pooled to achieve sufficient case numbers (labelled IPD-Work other).

Dementia was defined using ICD-10, codes F00, F01, F02, F03, G30 and G31 (31.0, 31.1, 31.8).

References: Peter R, Alfredsson L, Hammar N, Siegrist J, Theorell T, P. W. High effort, low reward, and cardiovascular risk factors in employed Swedish men and women: baseline results from the WOLF Study. *J Epidemiol Community Health* 1998; **52** :540-7

Alfredsson L, Hammar N, Fransson E, de Faire U, Hallqvist J, Knutsson A, et al. Job strain and major risk factors for coronary heart disease among employed males and females in a Swedish study on work, lipids and fibrinogen. *Scand J Work Environ Health* 2002; **28**: 238-48.

Whitehall II, the United Kingdom

The Whitehall II study is a prospective cohort study set up to investigate socioeconomic determinants of health. At study baseline in 1985-1988, 10 308 civil service employees (6 895 men and 3 413 women) aged 35-55 and working in 20 civil service departments in London were invited to participate in the study. Data on BMI from measured height and weight and the outcome measures from phase 3 were available for 8073 men and women who were eligible for our meta-analyses. The Whitehall II study protocol was approved by

the University College London Medical School committee on the ethics of human research. Written informed consent was obtained at each data collection wave.

Comprehensive tracing of electronic health records for dementia ascertainment was undertaken using three databases: the national hospital episode statistics (HES) database, the Mental Health Services Data Set (MHSDS) and the mortality register. Record linkage until 3Ist of March 2015, using International Classification of Diseases Tenth Edition (ICD-10) codes F00, F01, F02, F03, F05.1, G30, G31.0, G31.1 and G31.8 identified cases of dementia. The National Health Service (NHS) in the UK (England, Scotland, Wales) provides most of the health care, including out- and in-patient care. Private medical insurance, held by around 12% of the UK population (1997 figures),28 is mainly used for elective surgery rather than chronic conditions such as dementia. MHSDS is a national database which contains information for persons in contact with mental health services in hospitals, outpatient clinics, and the community. Mortality data were drawn from the British national mortality register (National Health Services Central Registry). The tracing exercise was carried out using the unique NHS identification number given to each resident in the UK.

Reference: Marmot MG, Davey Smith G, Stansfeld S, et al. Health inequalities among British civil servants: the Whitehall II study. *Lancet* 1991; **337**: 1387-93.

Webpage: https://www.ucl.ac.uk/whitehallII

***eTable 1*. Characteristics of the included cohort studies**

Study	Baseline	N(Total)	Mean age at BMI measurement	Proportion of women	Max follow-up, years	Person-years	N (Dementia)	Method of BMI measurement	Method of dementia ascertainment
FTC	1976	25,814	36.3	51.1	37.7	827,449	960	Measured	Deaths
Alameda	1965	6,861	43.7	54.2	35.9	193,935	35	Self-reported	Deaths
Nhis1986	1986	43,028	43.7	30.8	26.0	951,273	239	Self-reported	Deaths
Nhis1987	1987	85,100	44.0	46.3	25.0	1,822,167	474	Self-reported	Deaths
HALS	1984-1985	7,293	45.4	54.7	24.8	153,757	42	Measured	Deaths
Nhis1988	1988	85,140	44.3	46.4	24.0	1,763,067	489	Self-reported	Deaths
Whitehall II	1991 - 1993	8,064	49.6	30.8	23.9	177,486	239	Measured	Hospital & deaths
Nhis1989	1989	81,345	44.2	46.6	23.0	1,628,386	446	Self-reported	Deaths
Nhis1990	1990	83,459	44.3	46.5	22.0	1,613,081	425	Self-reported	Deaths
Nhanes1971	1971	14,396	48.9	59.7	21.4	260,397	102	Measured	Deaths
Nhis1991	1991	83,102	44.5	46.6	21.0	1,543,289	449	Self-reported	Deaths
Nhis1992	1992	88,060	44.4	46.7	21.0	1,639,100	447	Self-reported	Deaths
Hrs1992	1992	9,899	55.2	49.9	19.0	216,965	11	Self-reported	Deaths
Nhis1993	1993	75,781	44.6	46.8	19.0	1,291,418	364	Self-reported	Deaths
Nhanes3	1988-1994	18,072	46.8	53.2	18.1	238,438	86	Measured	Deaths
Nhis1994	1994	79,271	45.1	46.7	18.0	1,282,915	406	Self-reported	Deaths
WOLF_S	1992-1995	5,673	41.6	43.0	17.9	87,471	16	Measured	Hospital & deaths
Nhis1995	1995	68,721	44.6	46.8	17.0	1,063,138	299	Self-reported	Deaths
Nhanes2	1976-1980	9,246	54.3	53	16.8	122,842	16	Measured	Deaths
Nhis1996	1996	42,239	44.4	47.3	16.0	620,931	171	Self-reported	Deaths
Nhis1997	1997	33,523	46.1	56.4	15.3	465,807	154	Self-reported	Deaths
HeSSup	1998	23,894	36.7	59.0	15.0	352,597	81	Self-reported	Hospital, prescriptions, deaths
Nhis1998	1998	29,768	46.5	55.4	14.3	388,341	127	Self-reported	Deaths
Gazel	1997	11,342	50.3	27.6	14.0	154,810	14	Self-reported	Self-report, deaths
Nhis1999	1999	28,178	46.7	56.3	13.3	345,181	105	Self-reported	Deaths
Nhanes1999	1999-2000	4,900	46.3	53.1	12.8	53,590	21	Measured	Deaths
Nhis2000	2000	29,481	46.3	56	12.3	337,222	95	Self-reported	Deaths
FPS	2000	46,878	44.6	80.7	11.5	499,068	113	Self-reported	Hospital, prescriptions, deaths
Nhis2001	2001	30,218	46.3	55.6	11.3	319,395	90	Self-reported	Deaths
Nhanes2001	2001-2002	5,223	45.1	52.1	11.0	49,569	22	Measured	Deaths
Nhis2002	2002	27,736	46.7	55.4	10.3	268,815	88	Self-reported	Deaths
Nhanes2003	2003-2004	5,189	47.0	51.8	9.3	39,216	17	Measured	Deaths
Nhis2003	2003	27,153	46.7	55.7	9.3	238,863	66	Self-reported	Deaths
Nhis2004	2004	28,187	47.2	54.8	8.5	230,676	57	Self-reported	Deaths
Nhis2005	2005	28,053	47.5	55.3	7.3	195,895	52	Self-reported	Deaths
Nhanes2005	2005-2006	5,234	44.8	52	7.0	30,438	11	Measured	Deaths
Nhis2006	2006	22,312	47.0	54.8	6.3	135,425	20	Self-reported	Deaths
Nhis2007	2007	21,522	47.4	54.8	5.3	110,246	30	Self-reported	Deaths
Nhis2008	2008	20,502	47.9	55.5	4.3	85,435	15	Self-reported	Deaths
TOTAL	1965-2009	1,349,857				21,798,094	6894		

Appendix 2

Forest plots for meta-analysis of dementia risk per 5-unit increment in BMI after incremental exclusions to the follow-up

***eFigure 1*. Age-, sex-, and ethnicity-adjusted hazard ratio for dementia per 5-unit increment in BMI after no exclusions to the follow-up**

Study	N(Cases)	N(Total)	HR	(95 % CI)	p value
FTC	960	25814	1.11	(1.01–1.22)	0.0362
Alameda	35	6861	1.20	(0.80–1.80)	0.3681
Nhis1986	239	43028	0.92	(0.79–1.08)	0.3260
Nhis1987	474	85100	0.95	(0.85–1.06)	0.3720
HALS	42	7293	1.91	(1.42–2.56)	<.0001
Nhis1988	489	85140	0.85	(0.74–0.96)	0.0098
Whitehall II	239	8064	1.18	(1.01–1.38)	0.0432
Nhis1989	446	81345	0.92	(0.81–1.05)	0.3350
Nhis1990	425	83459	0.85	(0.74–0.97)	0.0147
Nhanes1971	102	14396	0.85	(0.68–1.07)	0.1659
Nhis1991	449	83102	0.93	(0.81–1.08)	0.3411
Nhis1992	447	88060	0.87	(0.77–0.98)	0.0238
Hrs1992	11	9899	0.62	(0.22–1.74)	0.3603
Nhis1993	364	75781	0.89	(0.76–1.04)	0.1387
Nhanes3	86	18072	0.96	(0.67–1.39)	0.8454
Nhis1994	406	79271	0.73	(0.64–0.84)	<.0001
WOLF_S	16	5673	0.78	(0.37–1.65)	0.5190
Nhis1995	299	68721	0.89	(0.77–1.03)	0.1262
Nhanes2	16	9246	0.51	(0.31–0.85)	0.0100
Nhis1996	171	42239	0.75	(0.59–0.95)	0.0153
Nhis1997	154	33523	0.74	(0.56–0.97)	0.0314
HeSSup	81	23894	0.82	(0.61–1.10)	0.1920
Nhis1998	127	29768	0.75	(0.61–0.92)	0.0062
Gazel	14	11342	0.87	(0.37–2.03)	0.7517
Nhis1999	105	28178	0.99	(0.77–1.26)	0.9069
Nhanes1999	21	4900	0.97	(0.61–1.54)	0.8842
Nhis2000	95	29481	0.80	(0.61–1.07)	0.1291
FPS	113	46878	0.81	(0.62–1.04)	0.0940
Nhis2001	90	30218	0.75	(0.57–0.98)	0.0356
Nhanes2001	22	5223	0.47	(0.27–0.83)	0.0091
Nhis2002	88	27736	0.73	(0.54–0.99)	0.0452
Nhis2003	66	27153	0.64	(0.45–0.92)	0.0170
Nhanes2003	17	5189	0.76	(0.43–1.34)	0.3370
Nhis2004	57	28187	1.04	(0.77–1.42)	0.7935
Nhis2005	52	28053	0.67	(0.44–1.01)	0.0578
nhanes2005	11	5234	0.67	(0.33–1.33)	0.2525
Nhis2006	20	22312	0.78	(0.45–1.33)	0.3569
Nhis2007	30	21522	0.35	(0.19–0.66)	0.0011
Nhis2008	15	20502	1.30	(0.92–1.82)	0.1343
Random effects model	6894	1349857	0.87	(0.82–0.93)	<.0001

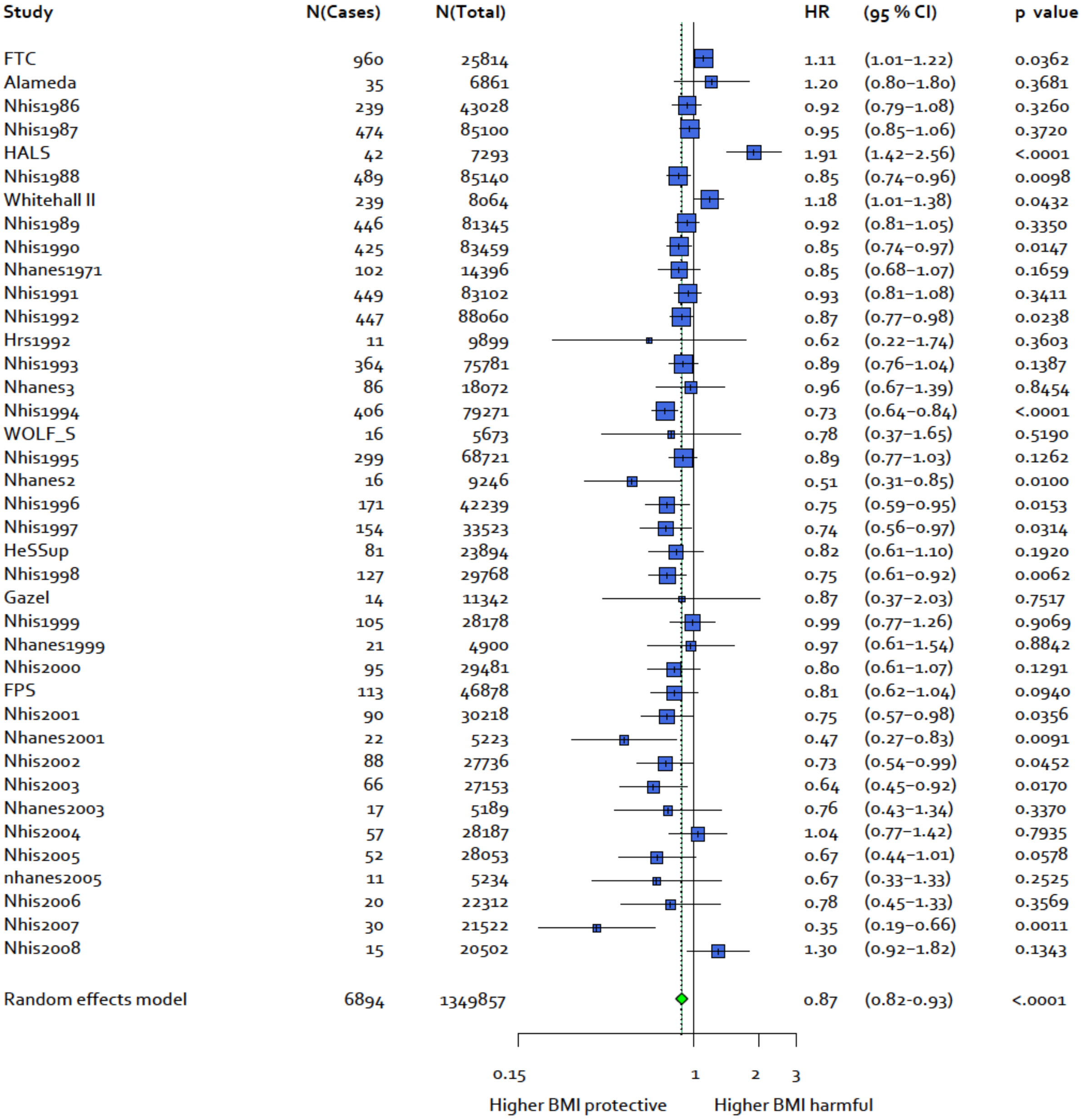

***eFigure 2*. Age-, sex-, and ethnicity-adjusted hazard ratio for dementia per 5-unit increment in BMI after excluding the first 5 years of the follow-up**

Study	N(Cases)	N(Total)	HR	(95 % CI)	p value
FTC	960	25069	1.10	(1.00–1.22)	0.0459
Alameda	35	6524	1.20	(0.80–1.80)	0.3681
Nhis1986	223	40753	0.95	(0.80–1.12)	0.5380
Nhis1987	457	80653	0.98	(0.88–1.09)	0.6593
HALS	41	6896	1.89	(1.40–2.55)	<.0001
Nhis1988	469	80706	0.85	(0.75–0.97)	0.0173
Whitehall II	238	7981	1.18	(1.01–1.38)	0.0425
Nhis1989	429	77132	0.93	(0.82–1.06)	0.2981
Nhis1990	408	79197	0.85	(0.75–0.98)	0.0204
Nhanes1971	100	13536	0.87	(0.69–1.10)	0.2524
Nhis1991	427	78831	0.95	(0.83–1.10)	0.5267
Nhis1992	419	83558	0.91	(0.81–1.02)	0.1068
Hrs1992	11	9426	0.62	(0.22–1.74)	0.3603
Nhis1993	343	71904	0.91	(0.78–1.07)	0.2635
Nhanes3	74	16584	1.06	(0.70–1.60)	0.7784
Nhis1994	387	75069	0.74	(0.64–0.84)	<.0001
WOLF_S	15	5591	0.85	(0.40–1.81)	0.6754
Nhis1995	279	65283	0.92	(0.79–1.07)	0.2708
Nhanes2	16	8668	0.51	(0.31–0.85)	0.0100
Nhis1996	149	40179	0.74	(0.58–0.94)	0.0158
Nhis1997	139	31665	0.79	(0.59–1.06)	0.1182
Hessup	67	23682	0.96	(0.72–1.30)	0.8113
Nhis1998	110	28053	0.80	(0.65–1.00)	0.0451
Gazel	13	11230	0.81	(0.33–1.97)	0.6351
Nhis1999	86	26620	1.03	(0.81–1.31)	0.8113
Nhanes1999	18	4607	1.21	(0.76–1.92)	0.4318
Nhis2000	71	27927	0.94	(0.71–1.25)	0.6704
FPS	89	46570	0.81	(0.61–1.08)	0.1447
Nhis2001	69	28568	0.80	(0.58–1.10)	0.1619
Nhanes2001	15	4952	0.41	(0.20–0.82)	0.0113
Nhis2002	56	26249	0.79	(0.55–1.15)	0.2151
Nhis2003	34	25671	0.76	(0.54–1.07)	0.1141
Nhis2004	37	26909	0.94	(0.62–1.43)	0.7741
Nhis2005	20	26563	0.69	(0.38–1.25)	0.2207
Random effects model	6304	1212806	0.92	(0.86–0.97)	0.0047

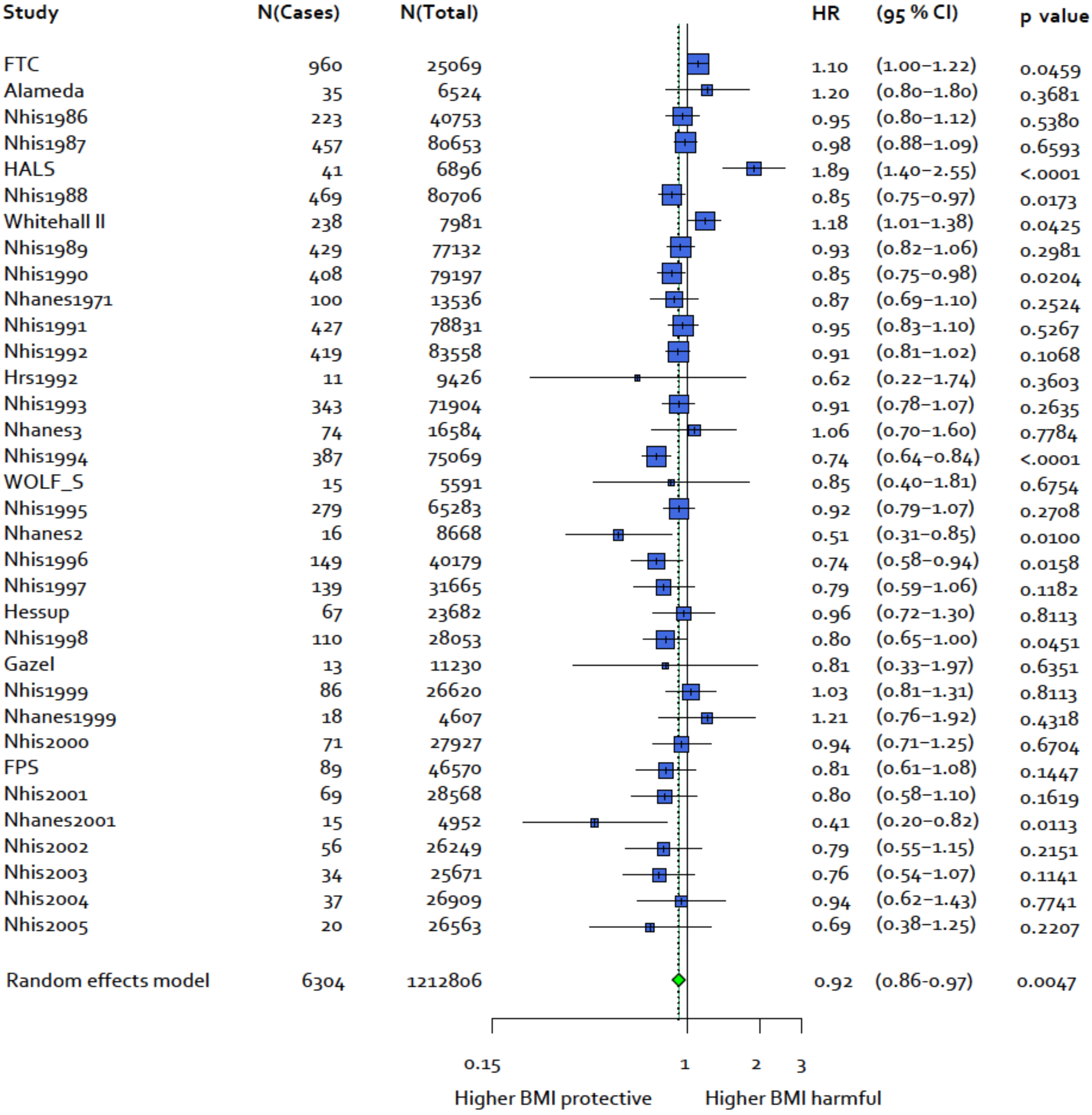

***eFigure 3*. Age-, sex-, and ethnicity-adjusted hazard ratio for dementia per 5-unit increment in BMI after excluding the first 10 years of the follow-up**

Study	N(Cases)	N(Total)	HR	(95 % CI)	p value
FTC	950	24292	1.11	(1.01–1.22)	0.0386
Alameda	35	6130	1.20	(0.80–1.80)	0.3681
Nhis1986	205	38194	0.96	(0.81–1.13)	0.6176
Nhis1987	420	75586	1.01	(0.90–1.12)	0.9094
HALS	35	6425	1.95	(1.43–2.67)	0.0000
Nhis1988	433	75651	0.88	(0.77–1.00)	0.0575
Whitehall II	234	7831	1.18	(1.01–1.39)	0.0394
Nhis1989	384	72262	0.94	(0.82–1.09)	0.4189
Nhis1990	358	74321	0.89	(0.78–1.02)	0.0891
Nhanes1971	91	12417	0.84	(0.66–1.07)	0.1633
Nhis1991	341	73995	0.99	(0.85–1.16)	0.9392
Nhis1992	321	78437	0.97	(0.85–1.11)	0.6647
Nhis1993	258	67494	0.91	(0.76–1.09)	0.3030
Nhanes3	44	15012	1.24	(0.75–2.05)	0.4094
Nhis1994	260	70243	0.86	(0.73–1.01)	0.0719
WOLF_S	14	5485	0.82	(0.37–1.80)	0.6180
Nhis1995	168	61317	0.93	(0.79–1.11)	0.4365
Nhanes2	13	7908	0.54	(0.31–0.92)	0.0228
Nhis1996	94	37846	0.79	(0.59–1.07)	0.1241
Nhis1997	85	29517	0.87	(0.59–1.30)	0.5083
HeSSup	44	23393	1.01	(0.70–1.45)	0.9597
Nhis1998	55	26093	1.12	(0.93–1.35)	0.2312
Gazel	11	11042	0.71	(0.26–1.96)	0.5128
Nhis1999	34	24777	1.12	(0.78–1.60)	0.5472
Nhis2000	16	26102	1.33	(0.79–2.22)	0.2800
FPS	21	38877	1.17	(0.71–1.94)	0.5316
Random effects model	4924	990647	0.99	(0.93-1.05)	0.7434

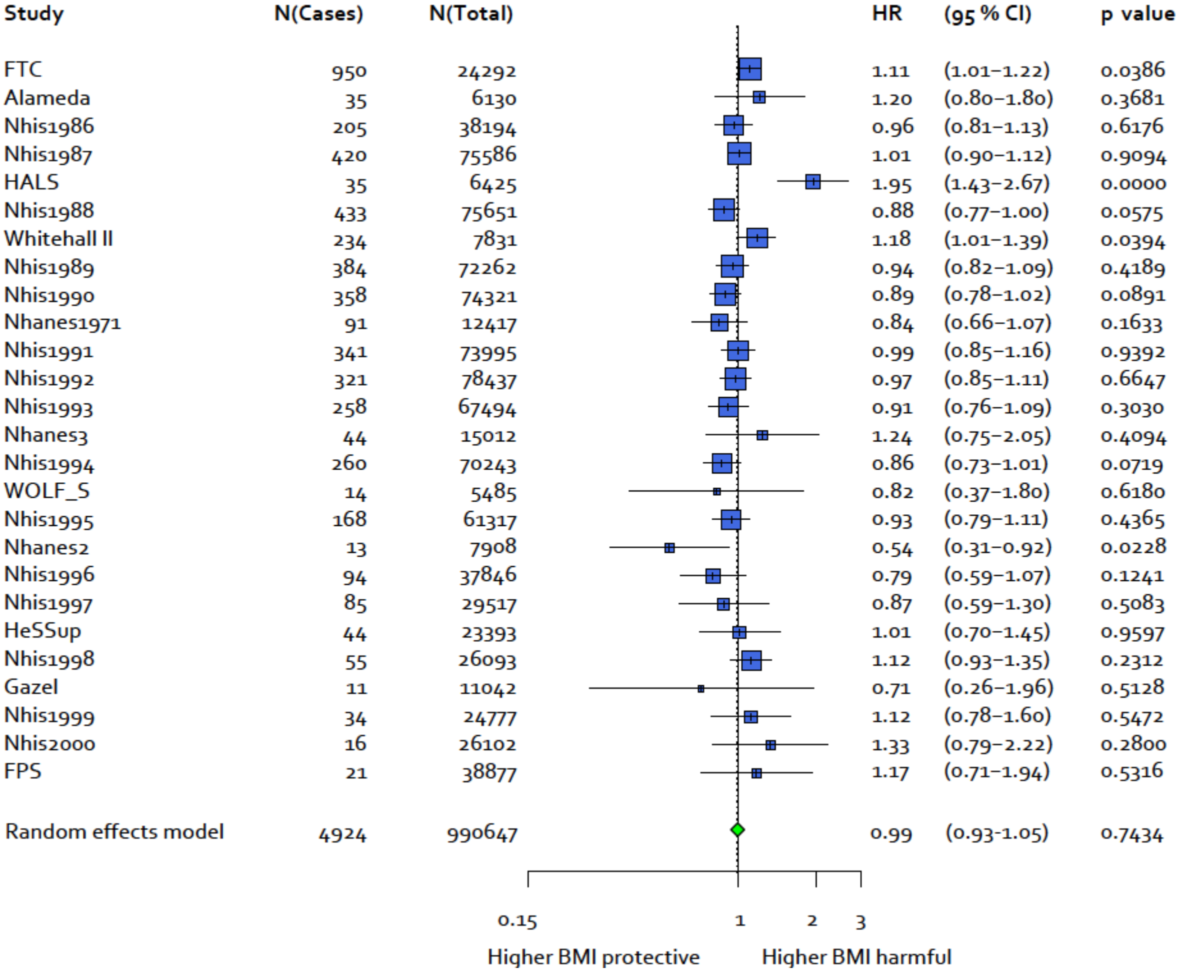

***eFigure 4*. Age-, sex-, and ethnicity-adjusted hazard ratio for dementia per 5-unit increment in BMI after excluding the first 15 years of the follow-up**

Study	N(Cases)	N(Total)	HR	(95 % CI)	p value
FTC	890	23264	1.13	(1.02–1.25)	0.0216
Alameda	35	5730	1.20	(0.80–1.80)	0.3681
Nhis1986	170	35517	0.99	(0.83–1.17)	0.8855
Nhis1987	327	70235	1.03	(0.92–1.16)	0.5941
HALS	27	5960	1.73	(1.18–2.54)	0.0051
Nhis1988	303	70254	0.95	(0.82–1.09)	0.4319
Whitehall II	217	7603	1.20	(1.02–1.42)	0.0289
Nhis1989	261	67282	1.04	(0.90–1.21)	0.5729
Nhis1990	214	69283	0.96	(0.81–1.13)	0.6078
Nhanes1971	60	11105	0.99	(0.75–1.32)	0.9626
Nhis1991	195	68943	1.03	(0.87–1.23)	0.7163
Nhis1992	162	73327	1.01	(0.84–1.21)	0.9444
Nhis1993	111	62983	0.98	(0.78–1.25)	0.8955
Nhis1994	82	65379	1.07	(0.83–1.38)	0.6086
Nhis1995	34	57360	1.29	(0.98–1.69)	0.0654
Random effects model	3088	694225	1.06	(1.00-1.11)	0.0394

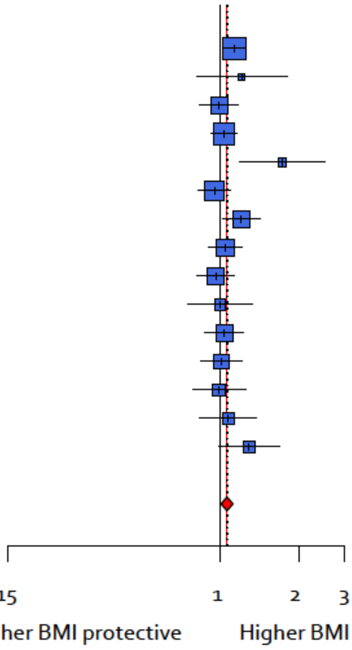

***eFigure 5*. Age-, sex-, and ethnicity-adjusted hazard ratio for dementia per 5-unit increment in BMI after excluding the first 20 years of the follow-up**

Study	N(Cases)	N(Total)	HR	(95 % CI)	p value
FTC	787	22084	1.12	(1.01–1.25)	0.0342
Alameda	33	5267	1.26	(0.84–1.89)	0.2555
Nhis1986	86	32818	1.13	(0.91–1.40)	0.2584
Nhis1987	161	64887	1.05	(0.88–1.25)	0.6087
HALS	14	5463	2.29	(1.45-3.61)	0.0004
Nhis1988	126	64952	0.99	(0.80–1.23)	0.9397
Whitehall II	122	7194	1.34	(1.09–1.65)	0.0052
Nhis1989	102	62317	1.17	(0.94–1.47)	0.1640
Nhis1990	53	64261	1.19	(0.90–1.58)	0.2157
Nhis1991	15	63951	0.92	(0.64–1.31)	0.6377
Random effects model	1499	393194	1.16	(1.05-1.27)	0.0030

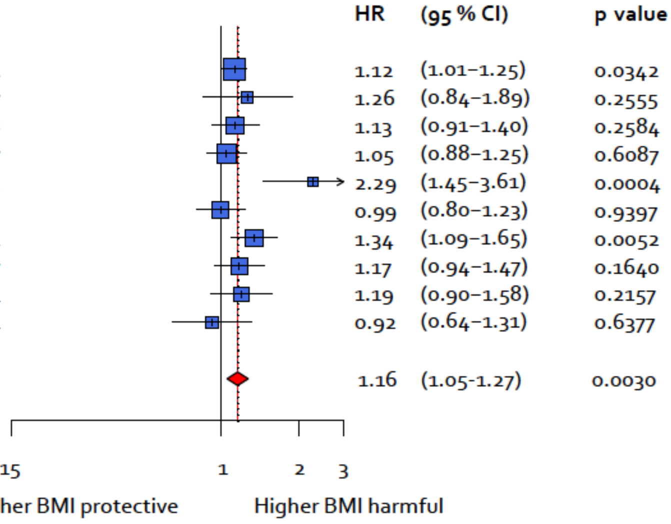

Appendix 3

Galbraith plots for the association between BMI and dementia after gradual exclusions to follow-up

***eFigure 6*. Galbraith plots for the association between BMI and dementia after gradual exclusions to follow-up**

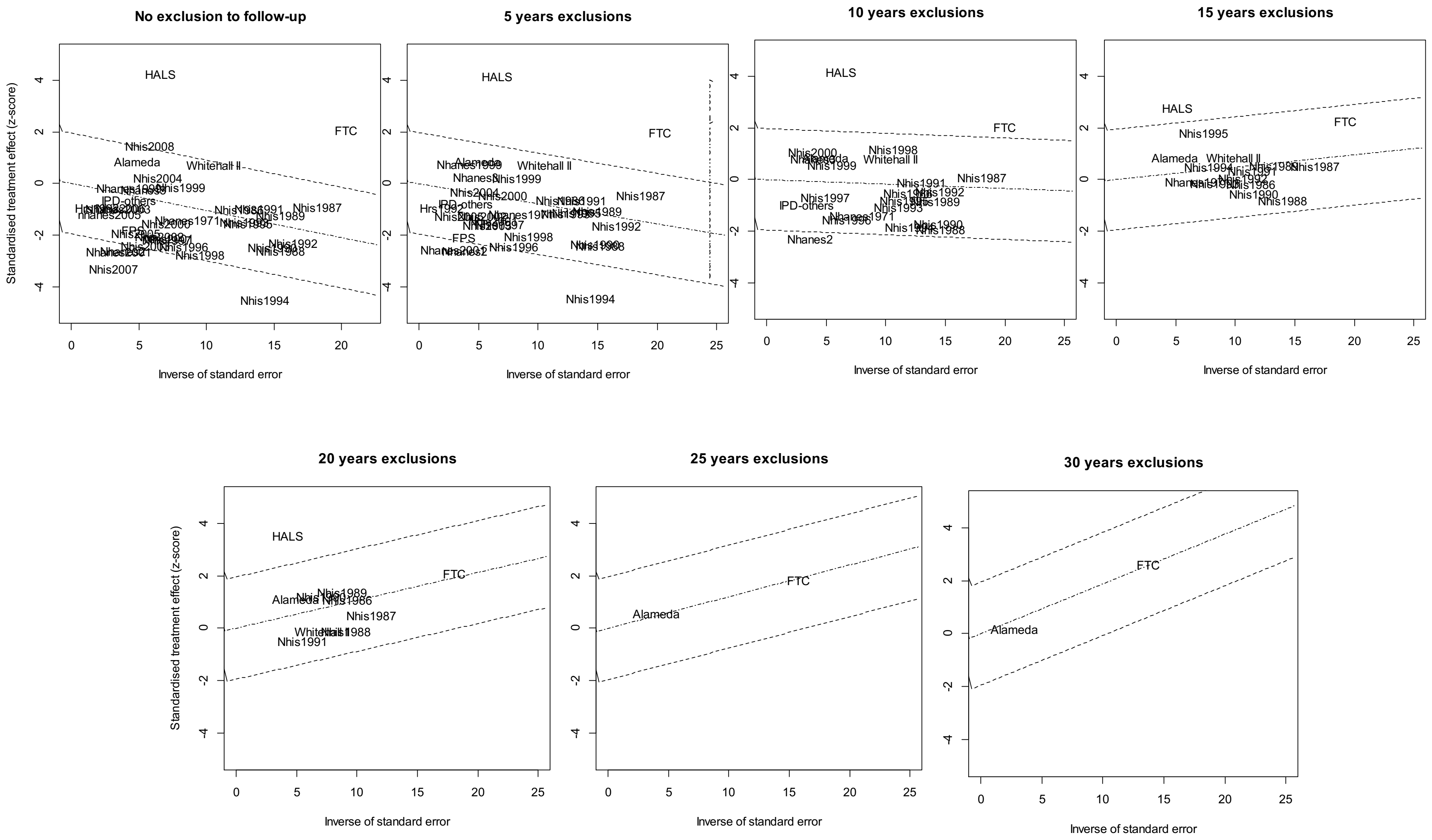

Appendix 4

Forest plots for meta-analyses of dementia risk per 5-unit increment in BMI stratified by the duration of follow-up

***eFigure 7*. Age-, sex- and ethnicity-adjusted hazard ratios for dementia risk per 5-unit increment in BMI stratified by the duration of follow-up**

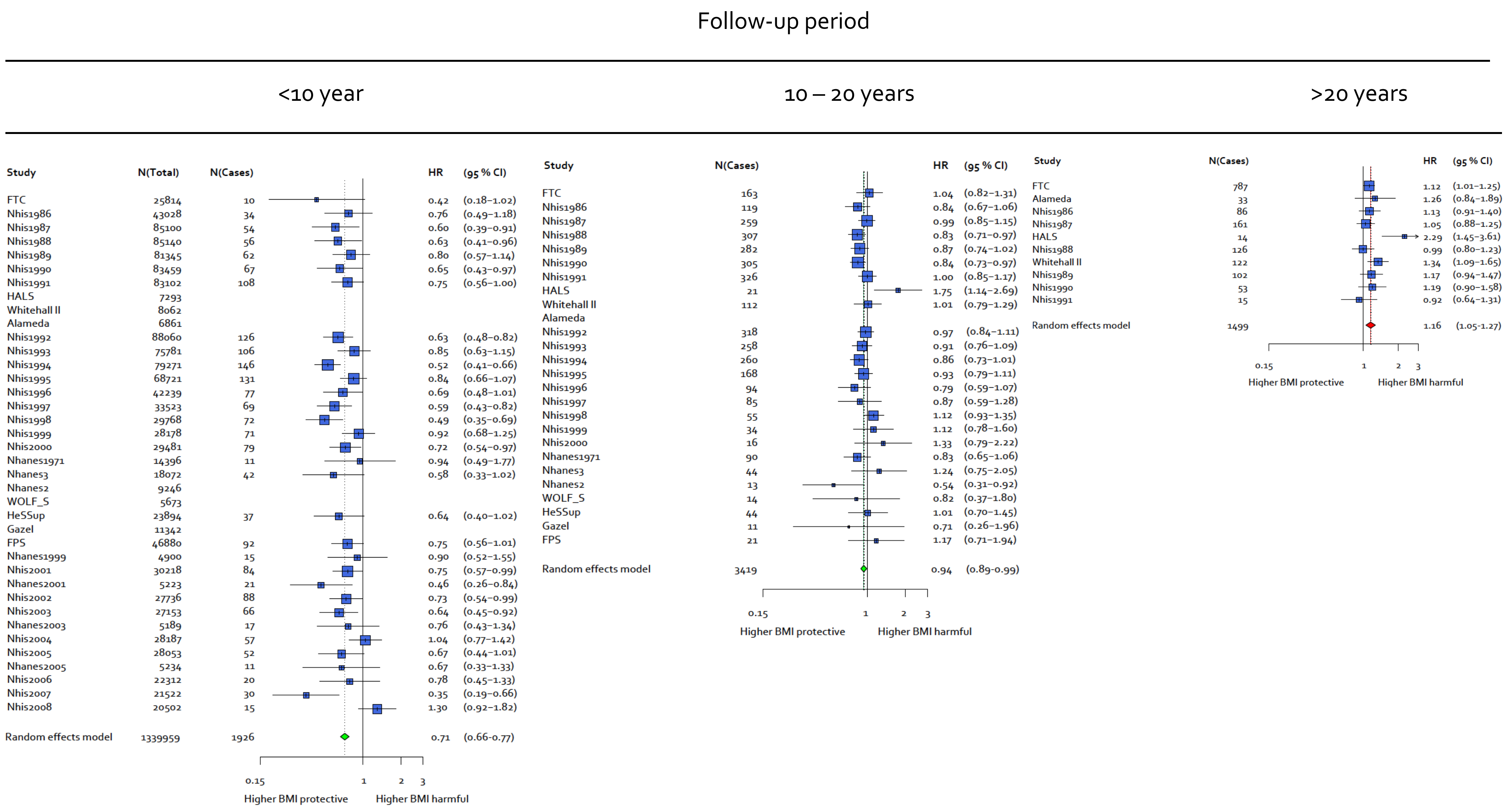

Appendix 5

Forest plots for meta-analyses of mortality risk before age 65 and after age 85 per 5-unit increment in BMI

***eFigure 8*. Age-, sex- and ethnicity-adjusted hazard ratio for the association between BMI (per 5-unit increment) and mortality before age 65**

Study	N(Cases)	HR	(95 % CI)	p value
FTC	2750	1.22	(1.15-1.30)	<.0001
Alameda	726	0.96	(0.87-1.06)	0.4163
Nhis1986	2992	1.10	(1.07-1.13)	<.0001
Nhis1987	5720	1.09	(1.06-1.12)	<.0001
HALS	390	1.04	(0.92-1.18)	0.5307
Nhis1988	5326	1.08	(1.05-1.11)	<.0001
Whitehall II	375	1.14	(1.02-1.28)	0.0239
Nhis1989	4908	1.10	(1.06-1.14)	<.0001
Nhis1990	4749	1.08	(1.05-1.11)	<.0001
Nhanes1971	843	1.10	(1.03-1.17)	0.0045
Nhis1991	4426	1.12	(1.09-1.15)	<.0001
Nhis1992	4553	1.10	(1.04-1.16)	0.0009
Hrs1992	817	1.16	(0.99-1.13)	0.0946
Nhis1993	3688	1.08	(1.05-1.11)	<.0001
Nhanes3	799	1.13	(1.05-1.22)	0.0011
Nhis1994	3673	1.09	(1.01-1.18)	0.0267
WOLF_S	126	1.33	(1.07-1.67)	0.0109
Nhis1995	3063	1.06	(1.02-1.10)	0.0030
Nhanes2	382	0.92	(0.80-1.06)	0.2423
Nhis1996	1833	1.06	(1.01-1.11)	0.0181
Nhis1997	1299	1.10	(1.04-1.16)	0.0009
HeSSup	751	1.17	(1.07-1.27)	0.0005
Nhis1998	1157	1.04	(0.98-1.10)	0.1958
Gazel	468	1.04	(0.91-1.20)	0.5592
Nhis1999	981	1.04	(0.98-1.10)	0.1958
Nhanes1999	144	0.96	(0.84-1.10)	0.5490
Nhis2000	950	1.09	(1.02-1.16)	0.0109
Nhis2001	858	1.09	(1.02-1.16)	0.0109
Nhanes2001	137	1.09	(0.96-1.24)	0.1835
FPS	764	1.24	(1.14-1.34)	<.0001
Nhis2002	716	1.07	(1.00-1.14)	0.0500
Nhis2003	646	1.14	(1.05-1.24)	0.0018
Nhanes2003	102	1.05	(0.90-1.22)	0.5350
Nhis2004	546	1.12	(1.02-1.23)	0.0175
Nhis2005	544	1.06	(0.96-1.17)	0.2491
Nhanes2005	81	0.96	(0.81-1.14)	0.6377
Nhis2006	360	1.10	(0.99-1.22)	0.0762
Nhis2007	272	0.96	(0.82-1.12)	0.6117
Nhis2008	209	1.11	(0.96-1.28)	0.1589
Random effects model	63124	1.09	(1.07-1.10)	<.0001

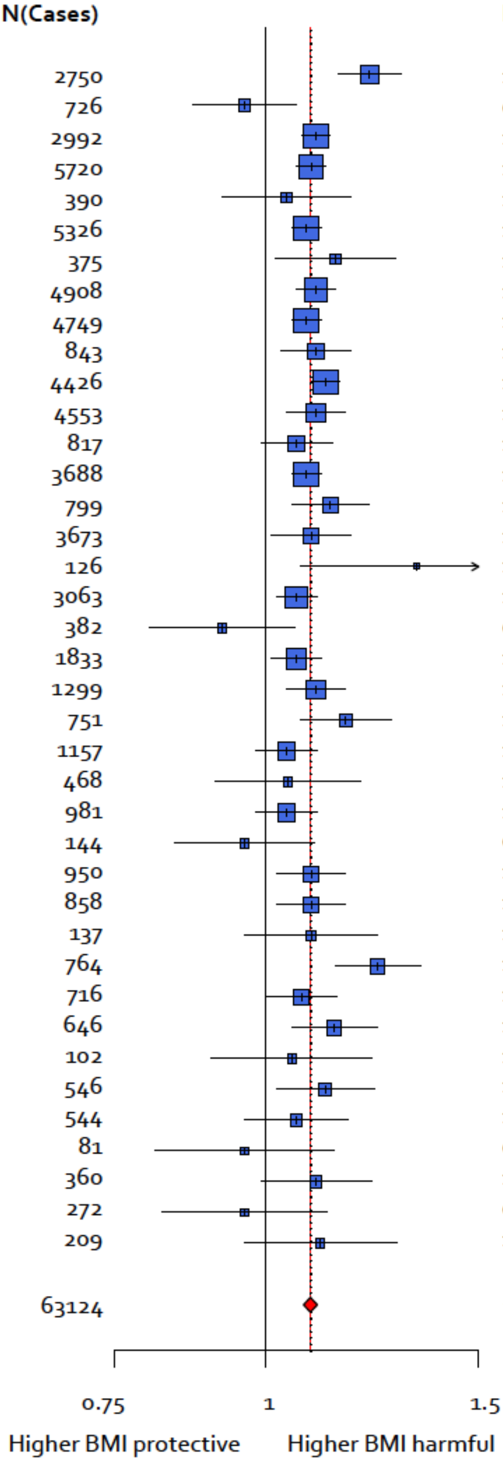

***eFigure 9*. Age-, sex- and ethnicity-adjusted hazard ratio for the association between BMI (per 5-unit increment) and mortality after age 85**

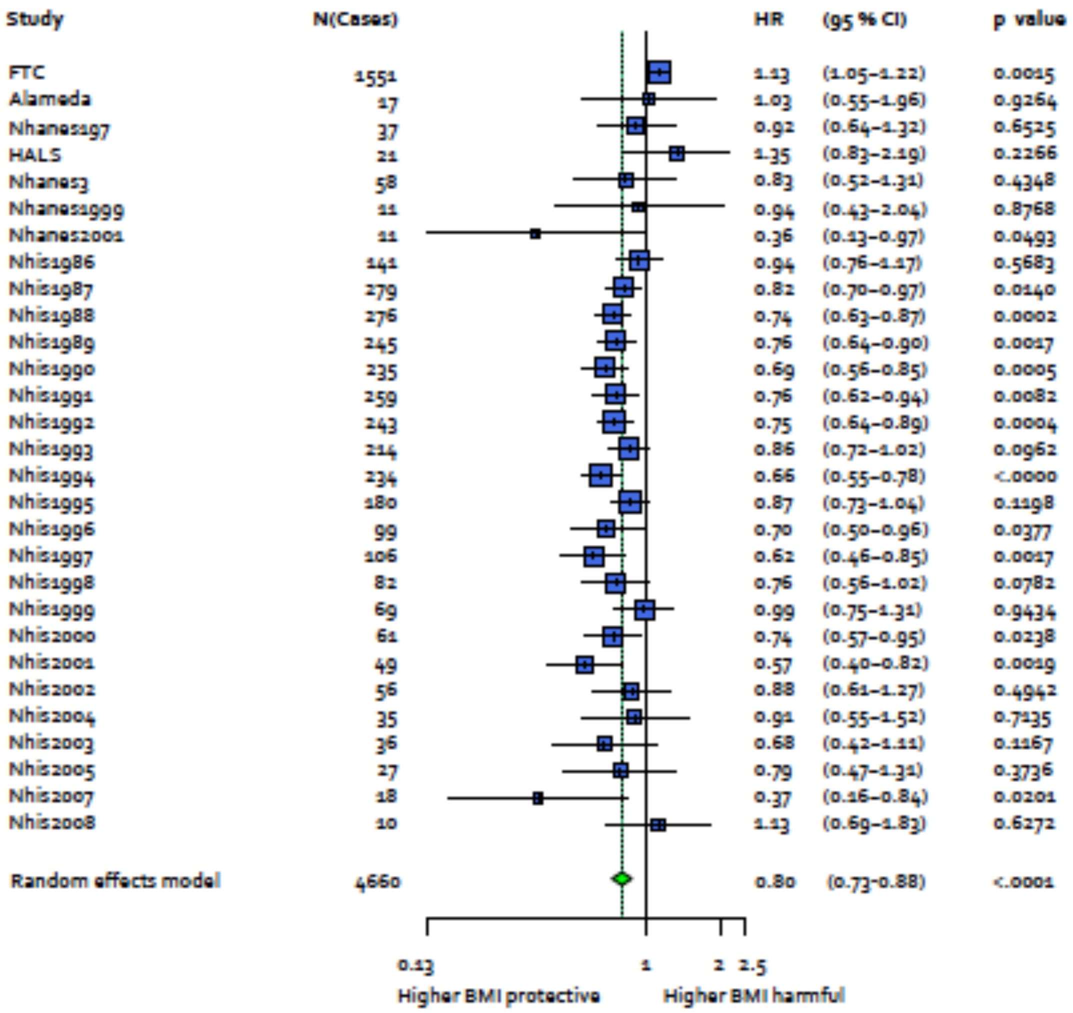

Appendix 6

Study-specific estimates for the association between BMI and dementia after excluding dementia cases ascertained from death registries.

***eTable 2*. Study-specific estimates for the association of BMI per 5-unit increase with dementia after excluding dementia cases ascertained from death registries in analyses with serial exclusions to the follow-up**

Study	N (cases)	N (total)	Hazard ratio (95% CI)	P-value
**No exclusions**				
Whitehall II	224	8064	1.15 (0.98 – 1.36)	0.0907
WOLF S	10	5673	0.55 (0.20 – 1.51)	0.2457
HeSSup	79	23,894	0.83 (0.62 – 1.11)	0.2110
GAZEL	12	11,342	1.00 (0.41 – 2.43)	0.9997
FPS	112	46,878	0.81 (0.63 – 1.05)	0.1108
Total	437	95,851	0.92 (0.74 – 1.15)	0.475
I^2= 51.7%, P=0.0816				
**5 years excluded**				
Whitehall II	223	7981	1.15 (0.98 – 1.36)	0.0894
WOLF S	9	5591	0.62 (0.22 – 1.75)	0.3664
HeSSup	67	23,682	0.96 (0.72 – 1.30)	0.8113
GAZEL	12	11,230	1.00 (0.41 – 2.43)	0.9997
FPS	88	46,570	0.82 (0.62 – 1.09)	0.1714
Total	399	95,054	0.99 (0.84 – 1.18)	0.947
I^2=25.8%, P=0.2493				
**10 years excluded**				
Whitehall II	220	7831	1.15 (0.98 – 1.36)	0.0929
WOLF S	8	5485	0.55 (0.18 – 1.69)	0.2983
HeSSup	44	23,393	1.01 (0.70 – 1.45)	0.9597
GAZEL	10	11,042	0.93 (0.34 – 2.55)	0.8863
FPS	21	38,898	1.17 (0.71 – 1.94)	0.5316
Total	303	86,649	1.11 (0.97 – 1.28)	0.139
I^2=0.0%, P=0.7110				
**15 year excluded**				
Whitehall II	205	7603	1.17 (0.98 – 1.39)	0.0776
WOLF S	3	4286	1.12 (0.22 – 5.59)	0.8917
Total	208	11,889	1.17 (0.98 – 1.39)	0.077
I^2= 0.0%, P=0.9581				
**20 years excluded**				
Whitehall II	118	7194	1.33 (1.08 – 1.65)	0.0081

***eTable 3*. Study-specific estimates for the association of BMI per 5-unit increase with dementia after excluding dementia cases ascertained from death registries in analyses stratified by follow-up period**

Study and follow-up	N (cases)	N (total)	Hazard ratio (95% CI)	P-value
**<10 years**				
Whitehall II	4	8064	1.14 (0.31 – 4.21)	0.8465
WOLF S	2	5673	0.55 (0.05 – 5.83)	0.6199
HeSSup	35	23,894	0.61 (0.37 – 1.00)	0.0497
GAZEL	2	11,342	1.30 (0.22 – 7.64)	0.7695
FPS	91	46,878	0.73 (0.55 – 0.98)	0.0384
Total	134	95,851	0.72 (0.56 – 0.92)	0.008
I^2= 0.0%, P=0.8429				
**10 – 20 years**				
Whitehall II	102	7831	0.95 (0.73 – 1.24)	0.6996
WOLF S	8	5485	0.55 (0.18 – 1.69)	0.2983
HeSSup	44	23,393	1.01 (0.70 – 1.45)	0.9597
GAZEL	10	11,042	0.93 (0.34 – 2.55)	0.8863
FPS	21	38,898	1.17 (0.71 – 1.94)	0.5316
Total	185	86,649	0.98 (0.81 – 1.19)	0.828
I^2=0.0%, P=0.8092				
**>20 years**				
Whitehall II	118	7194	1.33 (1.08 – 1.65)	0.0081
